# Expert and competent non-expert visual cues during simulated diagnosis in intensive care

**DOI:** 10.3389/fpsyg.2014.00949

**Published:** 2014-08-26

**Authors:** Clare McCormack, Mark W. Wiggins, Thomas Loveday, Marino Festa

**Affiliations:** ^1^Centre for Elite Performance, Expertise, and Training, Macquarie UniversityNorth Ryde, NSW, Australia; ^2^Paediatric Intensive Care Unit, Kim Oates Australian Paediatric Simulation Centre, Children’s Hospital at WestmeadWestmead, NSW, Australia

**Keywords:** expertise, cue utilization, diagnosis, medicine, simulation

## Abstract

The aim of this study was to examine the information acquisition strategies of expert and competent non-expert intensive care physicians during two simulated diagnostic scenarios involving respiratory distress in an infant. Specifically, the information acquisition performance of six experts and 12 competent non-experts was examined using an eye-tracker during the initial 90 s of the assessment of the patient. The results indicated that, in comparison to competent non-experts, experts recorded longer mean fixations, irrespective of the scenario. When the dwell times were examined against specific areas of interest, the results revealed that competent non-experts recorded greater overall dwell times on the nurse, where experts recorded relatively greater dwell times on the head and face of the manikin. In the context of the scenarios, experts recorded differential dwell times, spending relatively more time on the head and face during the seizure scenario than during the coughing scenario. The differences evident between experts and competent non-experts were interpreted as evidence of the relative availability of task-specific cues or heuristics in memory that might direct the process of information acquisition amongst expert physicians. The implications are discussed for the training and assessment of diagnostic skills.

## INTRODUCTION

The accurate initial assessment of clinical patients in time-critical emergencies is an essential component of timely and appropriate intervention by critical care teams (Pham et al., 2012). It mitigates the further deterioration of the patient’s condition and potentially reduces mortality and the additional burden on an already strained healthcare system. Nevertheless, it is a process that occurs within a short time-period and with potentially minimal information, thereby increasing the likelihood of error (Ely et al., 2011).

On the basis that assessments are required within a relatively short period and with minimal information, it is likely that a physician will engage lean and rapid cognitive strategies such as satisficing, relying on productions or relationships between patterns of information to guide the initial process of diagnosis (Simon, 1972; Marewski and Gigerenzer, 2012). Productions comprise rules-of-thumb or condition-action (IF-THEN) statements that are resident in memory and that can be used to assist the interpretation of a situation or event (Anderson, 1982; Hamm, 2014). For example, in the medical context, IF a patient presents with an elevated temperature, THEN it is normally associated with the presence of an infection.

The development and application of productions is generally associated with a reduction in cognitive load, since their application obviates the requirement for compensatory strategies that require the retention of task-related information in working memory (Sweller, 1988). However, such rules-of-thumb are, by definition, not necessarily applicable in all situations, and there are many cases where the application of productions has been associated with the commission of errors (Croskerry, 2003; Norman et al., 2014).

The acquisition of information as a prelude to the diagnosis of a particular condition is based, in part, upon the features that are immediately apparent on presentation to the physician (Croskerry, 2009a; Stolper et al., 2011). Where an association exists in memory, a feature or combination of features is presumed to trigger a production that will be interpreted as the basis of a diagnosis or will provide the impetus for the acquisition of additional information necessary to form a diagnosis (Khader et al., 2011). This process is consistent theoretically with the initial stages of recognition-driven decision-making where the condition-action statements that comprise productions are referred to as cues (Klein, 2008).

The acquisition and application of cues is thought to explain the rapid and consistently accurate behavior of genuine experts (Mann et al., 2007; Kahneman and Klein, 2009). In the context of the Recognition-Primed Decision model, cues trigger associations in memory that subsequently provide the basis for mental simulations that, in turn, guide a response (Klein, 2008). Brunswik (1955), in his Lens Model, also proposes that the likelihood of an association being triggered is dependent upon the frequency with which features in the environment match features in memory. Finally, Stokes et al. (1997) incorporate cues as the precursor to diagnosis in their theoretical model of expert decision-making in the aviation context.

Like productions, cues are essentially feature-event/object relationships in memory that enable the rapid assessment of a situation and, subsequently, the formulation of a response (Wiggins, 2006, 2012). Establishing the existence of cues has generally been inferred on the basis of responses to domain-specific stimuli. For example, Morrison et al. (2013) demonstrated that, in comparison to non-experts, expert forensic investigators were relatively consistent and responded more rapidly in assessing the relatedness of feature/event pairs relating to a murder investigation. Similarly, Wiggins and O’Hare (1995) established that the acquisition of weather-related information differed between experts and non-expert pilots, with the former being less likely to access information in the sequence in which it was presented. This behavior has been interpreted as evidence to suggest a greater level of cue utilization amongst experts.

The association between levels of cue utilization and expertise has been established in squash (Abernethy, 1990), power control (Loveday et al., 2013a), pediatric assessment (Loveday et al., 2013b), and aviation (Wiggins et al., 2014). Measures of cue utilization have also differentiated performance in the context of software engineering (Loveday and Wiggins, 2014). However, these approaches have been based on generalized behavior and there is no indication as to the specific cues involved and how they might be activated in response to the presence of features.

As experts gain experience within a particular context, Anderson (1982) suggests that productions are revised so that they become more precise and discriminate between different circumstances. Referred to as *discrimination*, it is a process that coincides with generalization where it becomes evident that a particular production is equally applicable across a range of conditions. This combination of discrimination and generalization may explain both the domain specificity of experts, together with their capacity to perceive underlying similarities between situations (Shanteau, 1988).

If experts possess a highly refined repertoire of task-related cues in memory, then the immediate features associated with two diagnostic scenarios that differ in their immediate features but incorporate a similar intrinsic etiology, should trigger the *bottom-up* application of distinct cues, and these differences should be evident in differences in the process of information acquisition (Patel and Groen, 1986; Croskerry, 2009b). Empirical support for this capacity for bottom-up discrimination can be drawn from research into the Einstellung Effect in which visual attention during expert problem-solving is implicitly drawn toward familiar solutions, even at the expense of novelty (Bilalić et al., 2010). Since competent non-experts have yet to develop highly specialized cues, they are not expected to alter their information acquisition in response to the differences in the immediate features of the task.

## MATERIALS AND METHODS

### PARTICIPANTS

The participants in the present study were drawn from a convenience sample of medical practitioners of different levels of grade and seniority, working in the pediatric and neonatal intensive care units of a tertiary children’s hospital. The local research and ethics committee approved the study, and individual participant consent was obtained from each clinician examined.

The participants comprised 11 male and seven female physicians employed in either pediatric or neonatal intensive care. Their mean age was 40.5 years, SD = 10.6. Establishing that expertise, rather than experience, has been acquired, requires that some formal criterion be established that is typically based on measures of performance. In the context of medical practitioners, an indirect measure of expertise is the seniority of their role (Patel and Groen, 1991). This constitutes recognition that they have successfully attained a level of performance where their medical interventions are both accurate and consistent over an extended period of time. While a positive relationship will inevitably exist between years of accumulated experience and performance, the recognition of expertise amongst peers presumes that a level of performance has been reached that is exceptional in comparison to other practitioners (Loveday and Wiggins, 2014). Therefore, consistent with this perspective, the participants were classified as expert or competent non-experts based on their occupational position (consultant/staff specialist, *n* =6, or trainee registrar/fellow, *n* =12) according to the criteria established by Patel and Groen (1991). Their accumulated experience working in medicine was between 6 and 42 years, *m* =16.5, SD = 10.6, with a range of 1–35 years in the intensive care environment, *m* =9.9, SD = 10.3. Experts recorded a mean 23.0 years experience working in intensive care, SD = 9.33, compared to a mean 4.8 years for competent non-experts, SD = 4.57.

### SIMULATION

**FIGURE 1:**
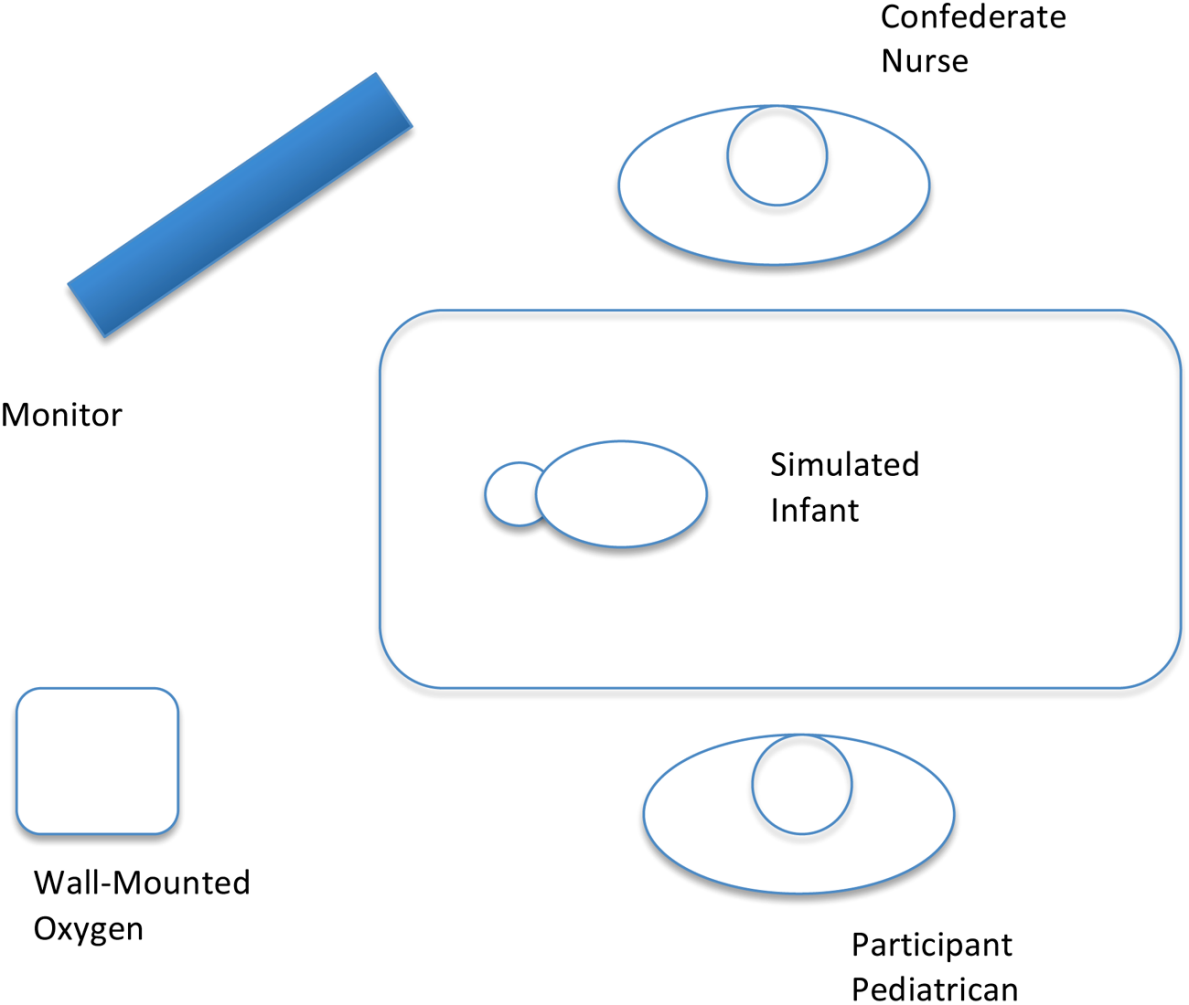
**Schematic showing the location of the various areas of interest (AoI), relative to the participant**.

A realistic scenario and naturalistic environment was created for the study by using a high-fidelity infant manikin (Laerdal SimBaby) connected to a monitor that displayed simulated physiological parameters and appropriate corresponding alarms and sounds *in situ* in an intensive care cot in a bedspace within the pediatric intensive care unit of a tertiary children’s hospital. The configuration of the room was typical of a bedspace in the pediatric intensive care unit and was familiar to all study participants (see **Figure 1**). A nasogastric feeding tube was inserted and attached to a continuous feeding pump with enteral feed attached. The manikin was also connected to nasal prong oxygen with a wall-mounted oxygen flow-meter, an intravenous drip via a peripherally inserted intravenous cannula, and an appropriately sized blood pressure cuff was attached to the right arm. A familiar and experienced pediatric intensive care nurse with a pre-scripted dialog was used as a confederate actor within the scenario.

An IVIEW X^TM^ HED eye tracking system manufactured by SensoMotoric Instruments was used to record the eye movements of participants, in addition to scene video and audio recording. The system consists of a fully mobile, head-mounted device with two cameras attached, one recording the scene and one trained on the participant’s eye, recording gaze and pupil data. A piece of clear plastic was fixed in front of one eye. The device was connected to a notebook computer, which powered the cameras and stored gaze, video and audio data. The gaze sampling rate used was 50 Hz, and a fixation was defined as 100 ms with maximum dispersion of 20 pixels. Based on the limitations imposed by frame rate acquisition and the need to include all features in the intensive care environments, features were broadly categorized as belonging to one of six areas of interest (AOI). Each area of interest was defined by anatomical or environmental relationships.

### SCENARIOS

The two scenarios used during the study were written by two subject-matter experts, both of whom were senior intensive care specialists working in the pediatric intensive care unit. The scenarios were designed around two immediate features, the first of which related to the head and face of the manikin. In particular, the level of consciousness of the child would be an important determinant in the seizure scenario, but would be less significant in the context of the coughing scenario. This information would be determined through the child’s facial features, including the eyes. In the coughing scenario, the information provided spontaneously by the assisting nurse was the immediate feature, since this would be an important determinant as to whether any respiratory assistance had been provided. Participants were randomly allocated to either the coughing or the seizure scenario as their first scenario, and all participants completed both scenarios.

The initial disease state was identical for both scenarios with the immediate features becoming evident as the symptomatology emerged. A simple respiratory arrest scenario in a self-ventilating monitored patient was used as the initial disease state, since it avoided potentially confounding effects that might be introduced by complex or unfamiliar equipment.

In the first minute of the coughing scenario, the patient demonstrated a heart rate of 150 beats per minute (BPM), blood pressure of 77/40 and a respiratory rate of 66 breaths per minute. Saturation was at 94% on 1 liter/min of nasal prong oxygen with good connections. This information, and electrocardiography (ECG), was displayed on the monitor. The patient showed see-saw breathing with bilateral crackles as well as grunting that was cycling with breaths. The cot was tilted at 30°. The scenario began with the nurse introducing the patient, saying: *“The ward is about ready to take this baby with bronchiolitis, but I’m concerned about whether he’s OK to be discharged from PICU as he’s had a couple of short desaturations as I’ve been looking after him this morning.”* They were also advised that the patient presented to the emergency department the previous evening with increased respiratory work, and was found to have respiratory syncytial virus (RSV) – positive bronchiolitis, and hyperinflation shown on a chest x-ray. The patient was admitted to PICU late on the previous afternoon for possible continuous positive airway pressure therapy, but improved with nasal oxygen. Feeds were started at 6.00am that morning, but had been stopped a few hours later following a second desaturation episode. Desaturations were associated with coughing and not with vomiting or the reflux of feed. No apnoea, bradycardia, or seizure was noted at the time. The temperature was at 37.6°C, and the patient was not on antibiotics. A pertussis swab had not been taken. A full blood count on admission showed hemoglobin (Hb) of 10.7 *g* per deciliter, white cell count (WCC) was 9.3 cells per cubic millimeter (Neutrophils 5.3, Leukocytes 4.0), and platelets at 210 cells per cubic milliliter.

After 1 min had elapsed, the manikin was set to cough for 20 s, desaturate to 84% over 40 s, and become bradycardic to 104 bpm over 40 s. At this point, the nurse prompted participants, saying: *“This is what he did before you came in.”* After 2 min and 10 s, saturation increased to 99% if the participant had used an oxygen bag, or to 94% if no adjustment to oxygen administration was made. Heart rate increased to 160 over 20 s, and the patient showed grunting and see-saw rasps as had occurred previously. The scenario concluded following a duration of 3 min and 30 s.

Prior to commencing the second scenario, participants were advised that this was a “new patient,” not related to the previous scenario. In the first minute of the seizure scenario, the patient had a heart rate of 120 bpm, blood pressure of 99/70 and respiratory rate of 33 breaths per minute. Saturation was at 94% on 1 liter/min of nasal prong oxygen with good connections. This information, and ECG, was displayed on the monitor. The patient showed see-saw breathing with bilateral crackles as well as grunting that was cycling with breaths. The cot was tilted at 30° and the scenario began with the nurse introducing the patient, saying: “*This baby has just been brought up from the ward by the nurse practitioner as he has had a couple of episodes of desaturation with stiffening of his arms and legs on the ward. I’m a little bit worried about him as he’s just had another similar episode and dropped his ‘sats’ to the mid 80 s. I’ve just done a capillary gas, which is in the gas machine now.”*

The participants were also advised that the patient was a 6 week old baby delivered at full term with no neonatal problems. The patient was presented to the ward 2 days previously with RSV positive bronchiolitis and hyperinflation shown on a chest x-ray. Since then, the patient had been on full maintenance intravenous fluids (N/4 and 5% dextrose) and nil by mouth. The patient was admitted to PICU an hour earlier. Since then, he had shown desaturation to the mid 85 associated with unusual movements of the torso and stiffening of limbs, and an increase in heart rate. The desaturations would self-correct after a minute of nasal prong oxygen, increased to 2 liter per minute. The temperature was at 37.6°C, and the patient was not on antibiotics. A pertussis swab had not been taken. A full blood count on admission showed Hb of 10.7 *g* per deciliter, WCC was 9.3 cells per cubic millimeter (Neutrophils 5.3, Leukocytes 4.0), and platelets at 210 cells per cubic milliliter.

After 1 min had elapsed, the manikin was programmed to show rapid and slow torso movements over 20 s, desaturation to 84% over 40 s and tachycardia to 180 over 40 s. At this point, the nurse prompted participants, saying: *“This is what he did before you came in. Here’s the cap gas* (hands over blood gas analysis).” After 2 min and 10 s, saturation increased to 99% over 20 s if the participant had used an oxygen bag, or to 94% if no adjustment to oxygen administration was made. Heart rate dropped to 160 over 20 s, and the patient showed see-saw rasps with the respiratory rate still at 33 breaths per minute. The scenario concluded following a duration of 3 min and 30 s.

### PROCEDURE

The participants completed a pre-scenario questionnaire that included demographic questions and questions related to participants’ subjective levels of fatigue and stress, and familiarity with the type of scenario encountered. The eye-tracker was then demonstrated to each participant, and the device fitted and calibrated using the recommended five-point calibration procedure.

Each participant took part in two consecutive scenarios separated by a 5 min interval. They waited outside the cubicle as the scenario was set up. The two scenarios were each of 3 min and 30 s duration and involved acute desaturation in a baby with bronchiolitis, due to either coughing (Scenario A) or a seizure/apnoea (Scenario B). A nurse was present in each scenario and briefed the clinician on the condition of the child over an equivalent period of time. The condition recovered spontaneously regardless of the treatment given.

Prior to each scenario, participants were reminded that they should regard the simulator as a real patient and that their individual performance was not being reported. The scenario began with the participant called to the bedspace by the confederate bedside nurse who introduced the scenario with a pre-scripted statement and a series of responses, and remained present throughout each scenario. Three researchers were also present in the cubicle during the study to monitor the eye-tracker, video-recording and simulator. All remained silent and out of view during the scenarios.

The eye-tracker automatically recorded eye movement data. Data for each participant were collated, including the number of fixations, the duration of fixations in milliseconds (dwell time), the number of blinks, the number of saccades, and the range of gaze. Video footage, taken from the perspective of participants, was also recorded throughout the tasks. The software package BeGaze^TM^ was used to align longitudinal data with video footage for the purposes of analysis. Video footage was analyzed frame by frame to identify AOI. There were six AOI defined in the visual scene, namely the nurse, the monitor, the manikin’s head and face, the manikin’s torso, the manikin’s limbs, and the wall on which the equipment and oxygen outlets were located.

## RESULTS

### DATA REDUCTION

To derive information on the process of visual information acquisition during initial clinical assessment, the video analysis was limited to the first 90 s of the scenario. Eye-tracking data for one expert and three competent non-experts were excluded from further analysis due to failed eye-tracking calibration. There was no airway opening, bag and mask support, or cardiac compression initiated by participants during the period of analysis.

### DESCRIPTIVE STATISTICS

Descriptive statistics were generated for each of the dependent variables. Across the participants and the scenarios, the mean dwell time was 448.16 ms, SD = 9.75. The mean dwell times for each of the AOI is summarized in **Table 1**.

**Table 1:** Mean dwell time (ms) by area of interest.

Area of interest	Mean	Std. error
Head	7183.407	1682.979
Torso	10203.959	2537.263
Limbs	374.330	143.209
Monitor	9628.809	1764.095
Nurse	4396.192	1633.480
Wall	1308.063	538.293

### FIXATIONS AND SACCADES

Three independent, mixed between-within analyses of variance were undertaken to establish whether a relationship existed between participants’ level of expertise, the nature of the scenario, and eye tracking behavior, including the frequency of fixations and saccades and the mean duration of fixations (dwell time).

No statistically significant differences were evident between experts and competent non-experts in the frequency of fixations, *F*(1,10) = 1.97, *p* = 0.19, η^2^ = 0.17, or saccades, *F*(1,10) = 4.00, *p* =0.07, η^2^ = 0.29. Similarly, eye gaze data were not significantly different between the scenarios in the frequency of fixations, *F*(1,10) = 3.89, *p* =0.07, η^2^ = 0.28, the frequency of saccades, *F*(1,10) = 2.46, *p*= 0.15, η^2^ = 0.20, or the mean dwell time, *F*(1,10) = 0.49, *p*= 0.50, η^2^ = 0.04.

Differences were, however, evident between experts and competent non-experts in the mean dwell time, *F*(1,10) = 6.48, *p = 0.03,* η^2^ = 0.39, with experts’ mean dwell time, 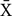 *=* 472.36 ms, SD = 14.89, greater than non-experts, 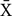 = 423.36 ms, SD = 12.58. No significant interaction was evident between expertise and scenario for the frequency of fixations, *F*(1,10) = 0.01, *p*= 0.91, η^2^ < 0.01, the frequency of saccades, *F*(1,10) = 0.04, *p*= 0.85, η^2^ < 0.01, nor the mean dwell time, *F*(1,10) = 0.58, *p*= 0.46, η^2^ = 0.05.

### AOI DWELL TIME ANALYSIS

**FIGURE 2:**
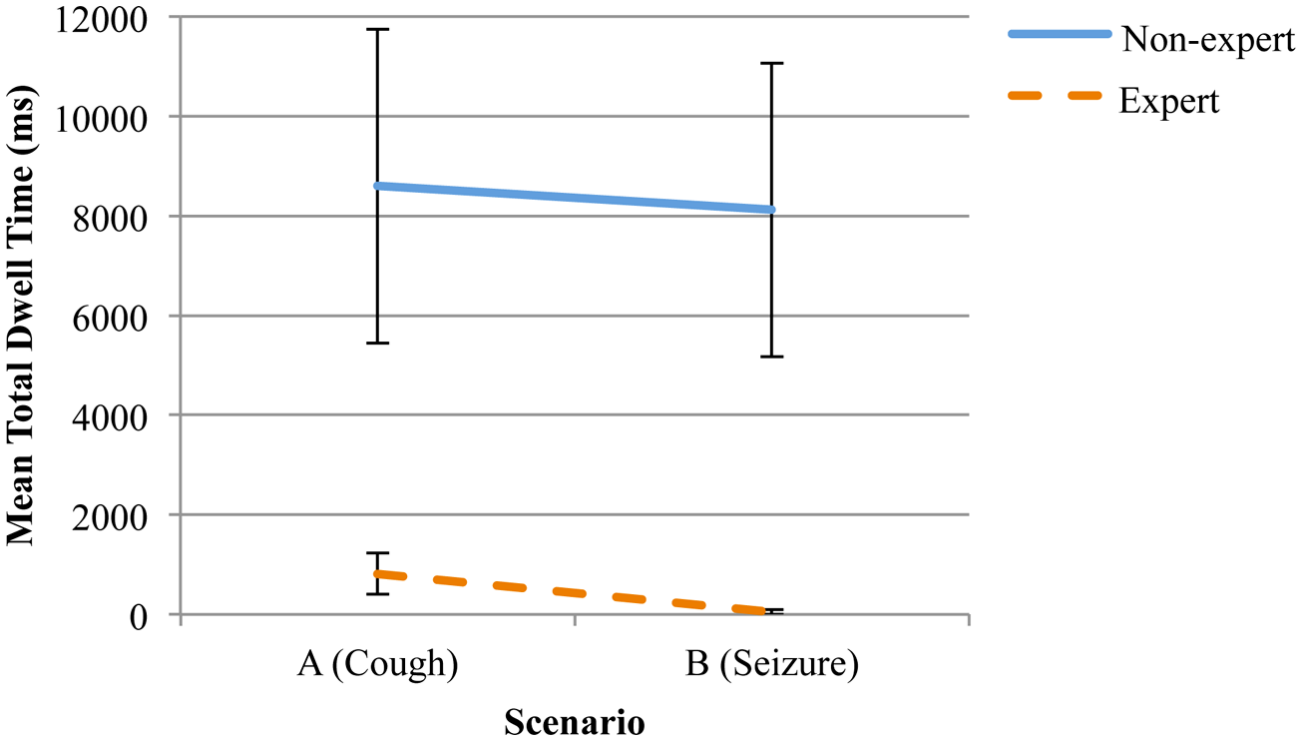
**Mean total dwell time (ms) and standard error on the “confederate nurse” for experts and competent non-experts, distributed across the two scenarios**.

For the “head and face” and “nurse” signature features, a 2 (expertise) × 2 (scenario) mixed-between ANOVA was used to test whether differences existed in the overall dwell time for experts and competent non-experts during the 90 s, initial assessment of the patient. It was assumed that differences in dwell time would reflect differences in the relative attention to features associated with the particular scenario. Consistent with expectations, the results revealed a significant difference between competent non-experts and experts’ mean overall dwell time for “head and face,” *F*(1,12) = 6.16, *p*= 0.03, η^2^ = 0.34, whereby experts recorded significantly greater dwell time within the AOI, 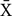 = 11358.83 ms, SD = 2698.77, in comparison to competent non-experts, 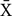 = 3007.98 ms, SD = 2011.55. A significant difference was also evident in the mean dwell time for the “nurse,” *F*(1,12) = 5.89, *p*= 0.03, η^2^ = 0.34. However, in this case, experts recorded a significantly lower dwell time within the AOI, 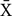 = 433.63 ms, SD = 2619.36, in comparison to competent non-experts, 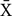 = 8358.76 ms, SD = 1952.38 (see **Figure 2**).

In the context of scenarios, a significant main effect was evident for the “head and face” AOI, *F*(1,12) = 5.69, *p*= 0.03, η^2^ = 0.32, whereby participants recorded a greater overall dwell time for this AOI during the seizure scenario, 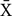 = 9438.06 ms, SD = 2452.35, in comparison to the coughing scenario, 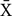 = 4928.75 ms, SD = 1199.43. This suggests that, as a cohort, both experts and competent non-experts responded to the differences between the scenarios by changing their pattern of information acquisition in relation to the head and face. However, there was no change evident in the overall dwell time on the nurse.

**FIGURE 3:**
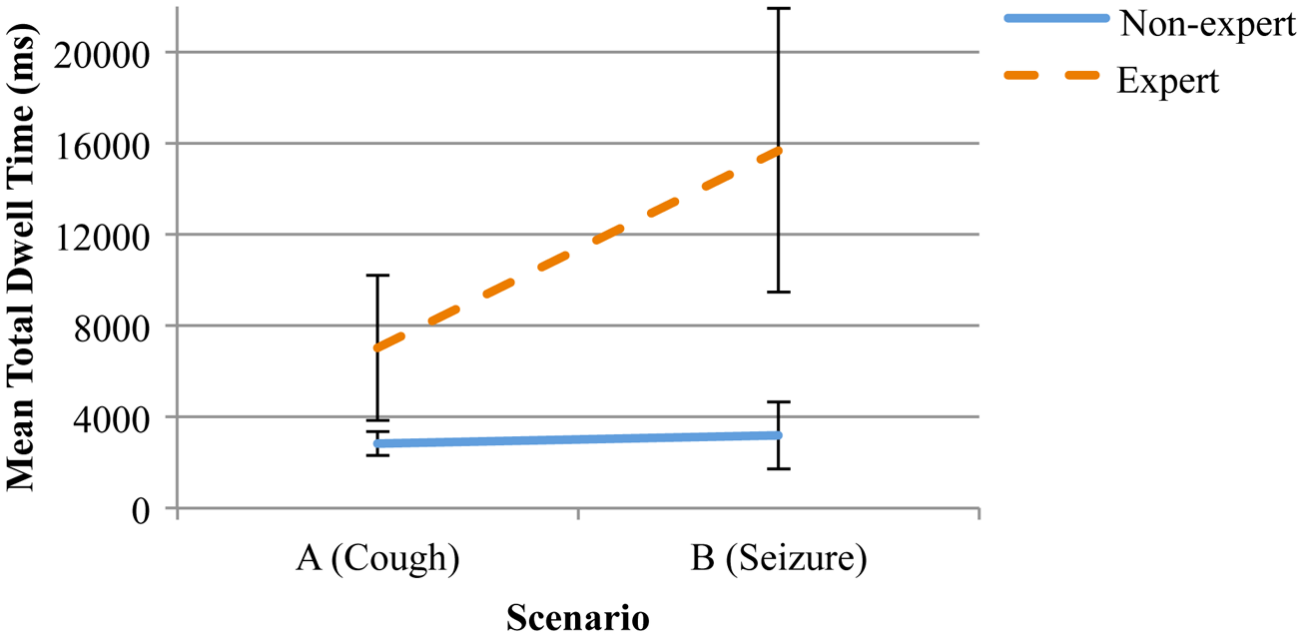
**Mean total dwell time (ms) and standard error on the “head and face” for experts and competent non-experts, distributed across the two scenarios**.

At a more detailed level, an expertise by scenario interaction was evident for the mean overall dwell time on the “head and face,” *F*(1,12) = 4.82, *p*= 0.04, η^2^ = 0.29. An inspection of the means indicated that, where there was relatively little difference between the mean dwell times for competent non-experts across the two scenarios, a difference was evident for experts with the mean dwell time greater during the seizure scenario than during the coughing scenario (see **Figure 3**). In combination, these results suggest that, although competent non-experts may recognize the relative importance of signature features during different diagnostic scenarios, their pattern of interaction with these features remains relatively consistent. This contrasts with expert clinicians who appear to alter both the overall time that they devote to the acquisition of information from signature features, together with the pattern of acquisition.

## GENERAL DISCUSSION

The aim of this study was to examine the information acquisition strategies employed by expert and competent non-expert intensive care physicians during two diagnostic scenarios that differed in their immediate features, but incorporated a similar intrinsic etiology. It was anticipated that where competent non-experts would adopt a relatively consistent pattern of information acquisition across the scenarios, experts would vary their approach consistent with the differences in the immediate features that were presented. The results revealed differences in overall mean fixation times between experts and competent non-experts, with the former maintaining visual gaze on AOIs for significantly longer periods. Further, experts spent significantly more dwell time within the “head and face” AOI and significantly less time within the “nurse” AOI in comparison to competent non-experts.

The differential performance amongst experts and competent non-experts during information acquisition is consistent with the proposition that experts and competent non-experts differ in their repertoire of cues in memory (Ericsson and Kintsch, 1995; Jarodzka et al., 2010). Experts attended to the immediate visual features associated with the patient (“head and face”), where competent non-experts tended to spend a greater proportion of the time fixated on the confederate nurse. Since this occurred independent of scenarios, it might be surmised that experts were integrating the auditory information being delivered by the confederate nurse with the visual information that was evident from the head and face of the manikin. It also implies that the “head and face” embodied a greater level of diagnostic information than was available from the nurse in isolation.

Despite the fact that, overall, experts tended to spend relatively more time than competent non-experts attending to the “head and face,” differences were evident in the mean dwell times across the scenarios. For competent non-experts, the relative emphasis on the “head and face” and “nurse” did not change with the change in scenario, suggesting that non-experts did not necessarily discriminate between the scenarios based on the immediate features.

As hypothesized, expert physicians recorded greater mean dwell times on the “head and face” during the seizure scenario, than during the coughing scenario. This reflects the potentially greater utility of the “head and face” in yielding diagnostic information during the coughing scenario. The dwell time for the “confederate nurse” did not change statistically for experts, possibly due to a restriction of range associated with the mean dwell times. In combination, the outcomes suggest that overall, experts spent more time examining cues arising from the “head and face” of the patient, but that differences in the immediate features were associated with differences in the time spent examining the cues.

Although the results confirm that experts differ from competent non-experts in their acquisition of information during diagnostic scenarios, it also suggests that their attention toward features in the environment is influenced by the interaction between their task-related experience and the immediate features that are present. For example, it is possible that, for competent non-experts, the situation was relatively unfamiliar and, therefore, they were seeking information that would correspond to a relatively limited number of patterns in memory. Since the “confederate nurse” was delivering an initial assessment of the symptoms, and may have experienced the event previously, directing attention toward the nurse represents a reasonable strategy, where a scenario is unfamiliar.

By contrast, experts possess a repertoire of cues in memory and therefore, are drawn toward features that are implicitly diagnostic of a particular condition (Croskerry, 2009b). The relative proportion of attention that is directed toward signature features is consistent with a bottom-up recognition process, whereby the environmental features trigger associations in memory, and a serial process of pattern matching is undertaken until a corresponding (or near to corresponding) pattern is identified (Patel and Groen, 1986; Klein, 2008).

At an applied level, the results suggest differences in the diagnostic strategies employed by experts and competent non-experts, and there are implications for training. For example, the fact that competent non-experts tended to attend to the nurse, suggests that they lacked a repertoire of cues in memory, necessary to recognize and adapt to the differences in the immediate features that were presented. This was not the case for experts who were able to identify the immediate features associated with the different scenarios and respond appropriately. One approach to the development of cues in memory involves cue-based training in which learners participate in a series of scenarios, the aim of which is to establish the relationship between features and events/objects in memory in the form of cues (Wiggins and O’Hare, 2003). The utility of cue-based training has been established in other domains (e.g., Auditors), and may be appropriate for diagnostic tasks in the medical context (Earley, 2001).

### LIMITATIONS AND FUTURE RESEARCH

While a weakness of this study is the relatively limited number of participants, the fact that differences were observed between experts and competent non-experts in relation to dwell times points toward the underlying power of the effects that were observed. Moreover, the study demonstrated that, in naturalistic environments, where the number of features available is relatively constrained and where the least experienced operators are in fact competent, differences in information acquisition were evident.

Since the focus of this study was information acquisition behavior during the initial assessment of a potentially deteriorating patient, the complexity associated with therapeutic interventions was excluded. Nevertheless, it is possible that a more extended observation may have revealed new information in the attention to cues, and the interactions with auditory and tactile stimuli. While these stimuli, were experimentally controlled in the present study, future research should be directed toward examining the relative impact of communication, and the social processes that are engaged by different groups of physicians. This builds on the baseline data that has been established in the present study and contributes to a broader understanding of non-visual stimuli or cues, and the role of team and social interactions in the recognition of the deteriorating child by skilled clinicians.

## CONCLUSION

This study demonstrated differences in the information acquisition behavior of experts and competent non-experts during assessments of a deteriorating child during two *in situ* simulations. Compared to competent non-experts, experts attended to specific visual features for longer periods, and exhibited longer dwell times on the manikin’s “head and face,” particularly during the seizure scenario. By contrast, competent non-experts displayed longer dwell times on the “confederate nurse.” These results were interpreted as evidence of differences between experts and competent non-expert physicians’ diagnostic cues in memory. The methodology offers a potential framework to develop behavioral standards of cue acquisition and utilization that could ultimately be used for the assessment of the diagnostic performance of physicians, particularly in time-constrained situations.

## Conflict of Interest Statement

The authors declare that the research was conducted in the absence of any commercial or financial relationships that could be construed as a potential conflict of interest.
